# Validation of a new instrument to guide and support insanity evaluations: the defendant’s insanity assessment support scale (DIASS)

**DOI:** 10.1038/s41398-022-01871-8

**Published:** 2022-03-22

**Authors:** Giovanna Parmigiani, Gabriele Mandarelli, Paolo Roma, Stefano Ferracuti

**Affiliations:** 1 Department of Human Neurosciences, “Sapienza” University of Rome, Rome, Italy; 2 Section of Criminology and Forensic Psychiatry, University of Bari, Interdisciplinary Department of Medicine, Bari, Italy

**Keywords:** Scientific community, Diseases

## Abstract

The insanity defense represents one of the most controversial and debated evaluations performed by forensic psychiatrists and psychologists. Despite the variation among different jurisdictions, in Western countries, the legal standards for insanity often rely on the presence of cognitive and/or volitional impairment of the defendant at the time of the crime. We developed the defendant’s insanity assessment support scale (DIASS) based on a wide view of competent decision-making, which reflects core issues relevant to legal insanity in many jurisdictions. To assess the characteristics of the DIASS we asked 40 forensic experts (16% women; years of experience = 20.6 ± 12.9) to evaluate 10 real-life derived forensic cases with the DIASS; cases included defendants’ psychiatric symptom severity, evaluated through the 24-itemBrief Psychiatric Rating Scale (BPRS). Exploratory factor analysis by principal axis factoring was conducted, which disclosed a two-factor solution explaining 57.6% of the total variance. The DIASS showed a good internal consistency (Cronbach’s alpha = 0.86), and substantial inter-rater reliability (Cohen’s kappa = 0.72). The capacities analyzed through the DIASS were mainly affected by mania/excitement and psychotic dimensions in nonresponsible and with substantially diminished responsibility defendants, while by hostility and negative symptoms in responsible defendants. The DIASS proved to be an effective psychometric tool to guide and structure insanity defense evaluations, in order to improve their consistency and reliability.

## Introduction

The insanity defense is a legal construct that, in specific circumstances, exempts defendants affected by mental illness from being held accountable for their criminal behavior [1]. It is a defense that dates to ancient times, and some traces can be found in ancient Greek and Roman texts [2]. The Napoleonic code was the main point of reference for the whole continental European codification of the nineteenth century. The legal nature of the criteria underlying insanity has sources that are also historical and that in Italy has led to giving greater weight to the epistemic and inhibitory control components. The most acknowledged standards in the Anglo-American systems are the M’Naghten Rule^1^, and The Model Penal Code’s test, also known as the American Law Institute (ALI) standard [3] (see Table 1).

**Table 1:** Insanity defense standards.

Standard	Definition	Cognitive/volitional prong
M’Naghten rule	A defendant is not found responsible if, due to a mental disorder, he did “not know the nature and quality of the act he was doing; or if he did know it, that he did not know what he was doing was wrong”.	C
American Law Institute (ALI) standard	“A person is not responsible for criminal conduct if at the time of such conduct as a result of mental disease or defect he lacks substantial capacity either to appreciate the criminality (wrongfulness) of his conduct or to conform his conduct to the requirements of the law”.	C, V

Nowadays, insanity definition and the threshold for satisfying its legal criteria tend to vary depending on the jurisdiction. Yet, in Western countries, the legal standards for insanity often rely on the presence of defendants’ cognitive and/or volitional impairment at the time of the crime [2].

In Italy, forensic experts have three possible conclusions regarding defendants’ criminal responsibility: nonresponsible, with substantially diminished responsibility, and responsible^2^ [4]. The forensic psychiatric evaluation leading to criminal irresponsibility or substantially diminished responsibility must also detect a cause-effect association between infirmity and specific criminal behaviour. Expert consultants can be appointed by the judge if he/she decides to avail himself/herself of their help or at the same time they can be summoned by the prosecution or by the defense with the aim of assessing the criminal responsibility and social dangerousness of the offender. However, the judge can also decide not to rely upon experts’ opinions nor is obliged to follow their suggestions.

Despite the presence of established legal criteria, the reliability and objectivity of insanity evaluations have been widely questioned, because of the frequent disagreement among forensic experts regarding the same case [5, 6], the lack of standardized procedures to guide the forensic evaluation [7], and the presence of unintentional or cognitive biases [7–10]. In addition, several factors such as money, prestige, and the amount of public attention attracted by the case have been shown to have an influence on forensic experts’ decisional processes [11]. Another source or limit could be represented by the intrinsic limits of psychiatric diagnosis and the longitudinal variability of psychiatric symptoms [12]. Moreover, discrepancies between forensic reports may be accounted for also by the absence of biological markers available and the relative scarcity of reliable diagnostic tools to guide forensic evaluations. Finally, the dialectic of the criminal trial itself may play a role, with the possibility of the presence of different opinions from different parties which plead their case, which the court or jury weighs.

To overcome these limits, theoretical models, some practice guidelines and forensic assessment instruments have been developed to assist the criminal responsibility evaluation [1, 13–21]. Among the tools used to perform insanity evaluations, are included the “Mental State at the Time of the Offense Screening Evaluation” (MSE) [22], the “Rogers Criminal Responsibility Assessment Scales” (R-CRAS) [23, 24], the “Rating scale of criminal responsibility for mentally disordered offenders” (RSCRs) [25], and the “Criminal Responsibility Scale” (CRS) [26]. In addition to those previously mentioned, there is the “Defendant’s Insanity Assessment Support Scale” (DIASS) [21]. Differently from the MSE, which is structured to screen out defendants whose criminal conduct clearly was not caused by significant mental abnormality, and the RSCRs, which evaluates defendants’ cognitive and volitional dimension through a hypothetical scenario based on clinical vignettes, the R-CRAS, RSCRs, and DIASS evaluate defendants’ criminal responsibility associated with the alleged crime.

The aim of the present study was to validate the DIASS, to study its psychometric properties and its factorial structure.

## Materials and methods

The present study was conducted from February 2020 to June 2020 and involved the development of 10 hypothetical forensic cases, based on real forensic cases modified to ensure nonrecognizability. Each forensic case included a measure of the defendant’s psychiatric symptomatology at the time of the forensic evaluation through the Brief Psychiatric Rating Scale (BPRS, Expanded Version) [27]. It was chosen to describe the psychiatric symptoms at the time of the assessment, since a detailed description of the symptoms is not available at the time of the crime, at least not such as to be able to fill in a BPRS. Usually, psychiatric symptoms at the time of the crime must be deduced retrospectively by the forensic evaluator on the basis of several factors such as the defendant’s account of the offense, past and present behavior, the characteristics of the alleged crime, and the psychiatric history of the accused, the witness report, or by video recordings, where available. Consequently, we provided hypothetical forensic cases with information related to the state of mind at the time of the crime, which was inferable from the methods used and data available, reported in the historical descriptions. The BPRS scores were based on the information available including what the experts had reported in their forensic reports of the real forensic cases. We decided to use this methodology, despite its possible limitations, because the reports we used to develop the hypothetical forensic cases neither include a BPRS evaluation nor other psychiatric rating scales. By doing so we assumed that the psychopathological elements that the expert had decided to describe in the report and therefore to convey to the judge, were those that the expert had taken into account in the decision-making process. This method has already been used in another study by our group [28]. Five BPRS factor scores were calculated, i.e., manic/excitement, depression/suicidality, hostility, positive symptoms, and negative symptoms [29].

We then sent the 10 hypothetical forensic cases by email to 50 experts in forensic psychiatry and legal psychology from Northern, Central and Southern Italy, asking them to rate just 5 of them (chosen by us) with the help of the DIASS and to apply its decision-making model to reach their final judgement (nonresponsible, substantially diminished responsibility, responsible), while evaluating the remaining 5 cases as they used to.

An ad-hoc form was developed to collect data about the forensic evaluator, such as name and surname, age, sex, years of forensic experience, and specialty.

Forensic cases were emailed to each examiner together with a cover letter explaining the aim of the study. Each evaluator was told to imagine being the judge’s expert consultant and was reminded of the study up to a maximum of three times, 1 month, 2 months, and 3 months after the first email, respectively. Those evaluators who still failed to give feedback were considered nonresponders.

### The Defendant’s Insanity Assessment Support Scale (DIASS)

The DIASS is a tool that has been developed on the theoretical model of competent decision-making and reflects core issues relevant to legal insanity in many jurisdictions in Western Countries [21]. It is composed of 9 binary items (present/absent) grouped into 4 dimensions: “Knowledge/understanding of the crime” (3 items), “Appreciating of the crime” (1 item), “Reasoning” (3 items), and “Control of voluntary motor activity” (2 items). The first two dimensions refer to the “Epistemic component”, while the third and fourth dimensions refer to the “Control component”. A box at the end of the scale refers to the final judgements on the Epistemic and Control components, which are scored on a 3-point scale (Intact, partially compromised and compromised). All the items refer to the state of mind of the accused, reconstructed at the time of the crime. The DIASS is an instrument designed to support and guide the insanity evaluation and should be used only after having examined all the legal and health documentation of the defendant, as a framework during the clinical evaluation. The instrument does not produce a compute score, but assists forensic evaluators in identifying which capacities relevant to mental criminal responsibility were present at the time of the offense. For a deeper description of the instrument and its development see Parmigiani et al., 2019 [21].

### Forensic cases

Each forensic case described the defendant’s personal, medical, social, and employment history, family relationships, arrest behavior, and current mental status. A summary of the 10 hypothetical forensic cases is shown in Table 2 (The full text is available on the Supplementary Material data file). The severity of psychiatric symptoms was shown through the BPRS.

**Table 2:** Hypothetical forensic cases summary.

Case descriptions	Sentence
Case 1 Diagnosis: Schizophrenia The defendant was a 40-year-old man, who killed his father and hit his brother. In the days following the crime he appeared scarcely aware of what had happened and scarcely cooperative to the clinical interview. Of that day he only remembered that the world had become “different, empty”. He referred to previous delusions of thought control and auditory hallucinations.	Nonresponsible
Case 2 Diagnosis: Bipolar disorder The defendant was a 28-year-old man, who harassed and threatened his ex-girlfriend because he did not accept the end of their relationship. He reported discontinuity in the attendance of the local Drug Rehabilitation Service and in therapy assumption; he appeared alert and oriented in the three axes, with ideational poverty, combined with a fairly basic suspicion and a tendency to overinterpret. His mood was instable with marked lability and dysphoric notes.	Nonresponsible
Case 3 Diagnosis: Delusional disorder The defendant was a 45-year-old man who mistreated his wife and attempted to poison her. He was already followed from the mental health center for a delusional disorder, jealousy type. He referred, in the months prior to the crime, the autonomous suspension of the antipsychotic therapy. At the forensic evaluation he appeared oriented in the three axes; the speech was fluid and the mood dysphoric. He was convinced that his wife was cheating on him with many men and that she wanted to kill him.	Nonresponsible
Case 4 Diagnosis: Delusional disorder/schizophrenia The defendant was a 32-year-old man who was accused of poisoning his father and brother with arsenic in a premeditated way. At the forensic evaluation he appeared poorly cared for in appearance and personal hygiene; he was alert, oriented in the three axes. Facial mimicry was considerably reduced, he stared back at the evaluator just for brief moments, and presented a poorly represented non-verbal communication, marked by a condition of apathy. A delusional ideation with mystic-religious content emerged. The mood was in line, although nuanced notes of demoralization were appreciated. Affectivity was constricted, awareness of illness was limited.	Nonresponsible
Case 5: Diagnosis: Schizoaffective disorder The defendant was a 39-year-old woman, accused of the murder of her 3-month-old son. She referred to a first hospitalization at the age of 20 for attempted suicide followed by other hospitalizations in psychiatric settings. At the forensic evaluation she appeared poorly cared for in appearance and oriented in the three axes. A delusional ideation of persecutory type (she motivated the act in question as an attempt to save her child) emerged. The mood was deflated.	Nonresponsible
Case 6: Diagnosis: Other specified personality disorder, mixed personality features (Histrionic/Narcisistic) The defendant was a 45-year-old woman, who was accused of the attempted murder of a friend. Psychiatric familiarity was absent. At the forensic evaluation she was oriented in the three axes; she presented a manipulative attitude and a mimicry marked by sadness; her cognitive functions were well preserved. She reported that it was not her intention to attack the victim.	Responsible
Case 7: Diagnosis: Unspecified personality disorder, unspecified bipolar disorder The defendant was a 44-year-old woman who was accused of personal injury, private violence, resistance, and damage to a Public Official. The victim was a pregnant woman, and the reason was a parking fight. Soon after the fight the defendant, visited by the ambulance staff called by some passer-by, was involuntarily hospitalized for “psychomotor agitation”. She denied psychiatric familiarity; she reported the presence of panic attacks, depression and attention deficit. At the forensic evaluation she was free from psychic disturbances, but a state of hypervigilance and persecutory tendencies emerged.	Substantially diminished responsibility
Case 8 Diagnosis: Substance induced psychotic disorder in paranoid personality disorder The defendant was a 32-year-old man accused of having hit and killed his uncle with 8 downward blows in the chest. At the forensic evaluation emerged a delusional ideation with a persecutory background towards the victim who had allegedly harmed the defendant’s family. He was poorly cared for in appearance and personal hygiene, and oriented in the three axes. The attitude was hypervigil. He reported use of cannabinoids since adolescence, initially occasionally, in recent years daily. The affectivity was flattened, the mood was oriented in a depressive sense. The awareness of the disease was poor.	Substantially diminished responsibility
Case 9 Diagnosis: Epilepsy—Jacksonian motoric crises, right facial-brachial-crural type with secondary generalization The defendant was a 42-year-old man, accused of killing a man with 18 stab wounds along with his brother-in-law during a robbery. At the forensic evaluation, he appeared smart in appearance and personal hygiene. The state of consciousness was alert, oriented in the three axes. Absent anomalies in the concentration, perception and memory (despite the subject reporting that the latter is not always effective, having had episodes in which he found himself in places unknown to him without knowing how he got there). He denied his involvement in the murder.	Responsible
Case 10 Diagnosis: Borderline intellectual functioning and ōther specified personality disorder, mixed personality features (Borderline/Antisocial) with impulsive sadistic traits related to sexual themes, probably paraphilic The accused was a 49-year-old man who assaulted and attempted to kill a prostitute with a knife, with 3 cuts to the abdomen. At the forensic evaluation, he appeared quite smart in appearance. The state of consciousness was quantitatively alert; he was oriented in the three axes. A detached, elusive attitude emerged on many subjects, at times he was frankly reticent and oppositional, probably because of, at least in part, a basic suspiciousness of the defendant. The mimicry was rigid, and expressionless; the speech was fluid, non-spontaneous. The thought seemed rather poor and concrete, affective participation was scarce. There were no current explicit formal logical alterations of thought or of perception. A low propensity to adapt to the usual social rules emerged, together with a tendency to be pleased with the suffering of others. Invited to describe his experiences in the various circumstances in which in the past he found himself exercising violent acts on women, he said he felt pleasure and anger at the same time	Responsible

### Statistical analysis

Statistical analyses were performed with the Statistical Software for Social Sciences v. 20.0 (SPSS). The alpha value was set to 0.05, all tests were two-tailed.

We performed an exploratory factor analysis by principal axis factoring of the DIASS items through Eigenvalue method >1 in order to extract the factors, also observing the scree plot. We rotated the factors by an oblique rotation (direct oblimin), and analyzed the DIASS internal consistency through Cronbach’s alpha.

To investigate Inter-Rater Reliability (IRR) Cohen’s kappa statistics [30] were used; IRR was investigated by comparing every possible combination of pairs of raters, scoring the same forensic case. Landis and Koch [31], have arbitrarily defined intervals in Cohen’s kappa for inter-rater agreement, where 0–0.20 is considered as slight, 0.21–0.40 as fair, 0.41–0.60 as moderate, 0.61–0.80 as substantial, and 0.81–1 as almost perfect agreement.

Spearman’s correlation coefficient was used to investigate associations among the items of the DIASS and the psychopathological dimensions investigated through the BPRS.

## Results

The 10 hypothetical forensic cases were sent to a sample composed of 50 forensic experts, of which 80% (*N* = 40) replied. The characteristics of the sample are shown in Table 3.

**Table 3:** Forensic experts’ sample characteristics.

Age, years, M (SD)	52 ± 11.9
Women, *n* (%)	8 (16)
Years of experience, M (SD)	20.6 ± 12.9
Profession, *n* (%)	
Psychiatrist	30 (75)
Psychologist	5 (12.5)
Medico-legal experts^a^	3 (7.5)
Neuropsychiatrist	2 (5)

^a^These medico-legal experts were expert in forensic psychopathology.

The exploratory factor analysis by principal axis factoring disclosed a 2-factor solution with a different distribution of the items compared to the original scale (Table 4), explaining 57.6% of the total variance. The first DIASS factor, which we named “epistemic component” comprised five items referring to the knowledge /understanding of the crime context (A1), appreciation of the criminal behavior through the subjective moral standard (B1), and *reasoning* about the possibility of non-acting/alternative choices (C1), about consequences -pros and cons (C2) and integration of relevant information (C3). The second DIASS factor, named “control component” comprised three items dealing with the knowledge/understanding of the criminality of the act and moral standard (A3), the control of voluntary motor activity through the ability to program, organize, finalize the action (D2), and the ability to inhibit one’s own behavior (D1). Item A2 (*knowledge/understanding* of the nature of the criminal act) was dropped from the scale because it loaded on both factors. In Appendix A is reported the final structure of the DIASS.

**Table 4:** DIASS principal component analysis.

DIASS items	Epistemic component (factor 1)	Control component (factor 2)
B1. Subjective moral standard (Appreciation of the criminal behavior)	0.843	
A1. Crime context (Knowledge/Understanding)	0.745	
C1. About possibility of non-acting/alternative choices (Reasoning)	0.732	
C3. Integration of relevant information (Reasoning)	0.727	
C2. About consequences (pros and cons) (Reasoning)	0.607	
A2. Nature of the criminal act (Knowledge/Understanding)	0.419	0.413
D2. Ability to program, organize, finalize the action (Control of voluntary motor activity)		0.938
A3. Criminality of the act and moral standard (Knowledge/Understanding)		0.609
D1. Ability to inhibit one’s own behavior (Control of voluntary motor activity)		0.593
Variance explained	46.98	10.64

The DIASS showed good internal consistency (Cronbach’s alpha = 0.86), and a good concurrent validity as shown through the highly significant association between forensic experts’ judgment reached using the DIASS and the court ruling on the real cases (rho = 0.674; *p* < 0.001).

Cohen’s kappa disclosed substantial DIASS internal consistency among different raters varying from 0.44 to 1, with a mean value of 0.72.

No differences emerged between forensic experts’ final judgement reached with the use of the DIASS and without it, as disclosed by Fisher’s exact test (Table 5).

**Table 5:** Differences in forensic cases evaluation performed with and without the use of the DIASS.

	with DIASS	without DIASS	*p*
Forensic case *n* 1			
Nonresponsible, *n* (%)	17 (100)	23 (100)	ns
Substantially diminished responsibility, *n* (%)	0 (0)	0 (0)	
Responsible, *n* (%)	0 (0)	0 (0)	
Forensic case *n* 2			
Nonresponsible, *n* (%)	4 (23.5)	3 (13)	ns
Substantially diminished responsibility, *n* (%)	10 (58.8)	18 (78.3)	
Responsible, *n* (%)	3 (17.6)	2 (8.7)	
Forensic case *n* 3			
Nonresponsible, *n* (%)	9 (52.9)	13 (56.5)	ns
Substantially diminished responsibility, *n* (%)	6 (35.3)	8 (34.8)	
Responsible, *n* (%)	2 (11.8)	2 (8.7)	
Forensic case *n* 4			
Nonresponsible, *n* (%)	11 (64.7)	11 (47.8)	ns
Substantially diminished responsibility, *n* (%)	6 (35.3)	10 (43.5)	
Responsible, *n* (%)	0 (0)	2 (8.7)	
Forensic case *n* 5			
Nonresponsible, *n* (%)	15 (88.2)	23 (100)	ns
Substantially diminished responsibility, *n* (%)	2 (11.8)	0	
Responsible, *n* (%)	0 (0)	0	
Forensic case *n* 6			
Nonresponsible, *n* (%)	0 (0)	1 (5.9)	ns
Substantially diminished responsibility, *n* (%)	3 (13)	3 (17.6)	
Responsible, *n* (%)	20 (87)	13 (76.5)	
Forensic case *n* 7			
Nonresponsible, *n* (%)	8 (34.8)	7 (41.2)	ns
Substantially diminished responsibility, *n* (%)	12 (52.2)	7 (41.2)	
Responsible, *n* (%)	3 (13)	3 (17.6)	
Forensic case *n* 8			
Nonresponsible, *n* (%)	15 (65.2)	10 (58.8)	ns
Substantially diminished responsibility, *n* (%)	7 (30.4)	6 (35.3)	
Responsible, *n* (%)	1 (4.3)	1 (5.9)	
Forensic case *n* 9			
Nonresponsible, *n* (%)	0 (0)	0 (0)	ns
Substantially diminished responsibility, *n* (%)	1 (4.3)	2 (11.8)	
Responsible, *n* (%)	22 (95.7)	15 (88.2)	
Forensic case *n* 10			
Nonresponsible, *n* (%)	9 (52.9)	0 (0)	ns
Substantially diminished responsibility, *n* (%)	6 (35.3)	5 (29.4)	
Responsible, *n* (%)	2 (11.8)	12 (70.6)	

*p-*values by Fisher’s Exact Test.

*ns* not significant.

Correlations among the capacities analyzed through the DIASS and the severity of psychiatric symptoms in nonresponsible, with substantially diminished responsibility and responsible defendants are shown in Table 6.

**Table 6:** Correlations between the items of the DIASS and psychopathological dimension in defendant judged nonresponsible, with substantially diminished responsibility and responsible.

Nonresponsible					
	Mania/excitement	Depression/suicidality	Hostility	Positive symptoms	Negative symptoms
A1. Crime context	0.291**	0.234*	−0.171	−0.238*	−0.417**
B1. Subjective moral standard	0.031	0.129	−0.048	−0.082	−0.138
C1. About possibility of non-acting/alternative choices	−0.039	0.01	−0.030	−0.145	−0.207
C2. About consequences (pros and cons)	−0.119	−0.097	−0.164	−0.082	−0.194
C3. Integration of relevant information	−0.131	−0.090	−0.068	−0.074	−0.135
A3. Criminality of the act and moral standard	−0.172	−0.246*	−0.008	0.050	−0.149
D1. Ability to inhibit one’s own behavior	−0.236*	−0.067	−0.182	−0.097	−0.076
D2. Ability to program, organize, finalize the action	−0.406**	−0.025	−0.215*	−0.135	−0.152
Epistemic component	−0.119	−0.154	0.235*	0.211	0.346**
Control component	0.216*	0.054	0.134	0.126	0.222*
**Substantially diminished responsibility**					
A1. Crime context	−0.272*	−0.227	−0.163	−0.509**	−0.127
B1. Subjective moral standard	0.059	0.019	0.017	0.040	−0.098
C1. About possibility of non-acting/alternative choices	0.007	−0.140	0.130	0.034	0.087
C2. About consequences (pros and cons)	−0.070	−0.180	0.029	0.080	0.133
C3. Integration of relevant information	−0.198	−0.260*	0.116	−0.309*	−0.018
A3. Criminality of the act and moral standard	−0.030	0.024	0.133	−0.236	−0.248
D1. Ability to inhibit one’s own behavior	−0.041	−0.110	0.048	0.542**	0.456**
D2. Ability to program, organize, finalize the action	−0.049	−0.211	0.270*	0.119	0.238
Epistemic component	0.309*	0.208	0.049	0.406**	0.153
Control component	0.052	0.092	−0.115	−0.234	−0.227
**Responsible**					
A1. Crime context	0.056	−0.102	0.003	−0.100	0.082
B1. Subjective moral standard	−0.107	0.168	−0.455**	0.019	−0.484**
C1. About possibility of non-acting/alternative choices	−0.354**	0.036	−0.275*	−0.178	−0.238
C2. About consequences (pros and cons)	−0.165	−0.033	−0.305*	−0.116	−0.186
C3. Integration of relevant information	−0.083	0.238	−0.279*	0.149	−0.393**
A3. Criminality of the act and moral standard	−0.166	0.106	−0.312*	−0.144	−0.358**
D1. Ability to inhibit one’s own behavior	−0.370**	0.107	−0.411**	−0.093	−0.402**
D2. Ability to program, organize, finalize the action	−0.076	0.109	−0.129	0.074	−0.130
Epistemic component	0.263*	0.061	0.393**	0.165	0.301*
Control component	0.507**	−0.037	0.504**	0.172	0.483**

*p*-values by Spearman’s correlation coefficient.

*p < 0.05.

**p < 0.01.

## Discussion

Our study on a sample of 40 forensic experts confirmed the bifactorial structure of the DIASS, although exploratory factor analysis revealed a different distribution of the items than the original construct/scale. Specifically, item A3, (Knowledge/Understanding of the criminality of the act and moral standard), which was originally conceived as part of the epistemic component, after the exploratory factor analysis, loaded on the control component. In interpreting this result, we must consider that this item deals with an aspect that forensic experts analyze through the observation of external behavior, which is associated with the notion of inhibitory control. For example, the absence of efforts to avoid detection or apprehension may suggest a lack of knowledge/understanding of wrongfulness.

The DIASS showed good internal consistency, concurrent validity and substantial inter-rater reliability. These results underline the good psychometric characteristics of the DIASS as well as the importance for criminal responsibility evaluations of both the cognitive and control components, as already is the case in Italy and in those countries, which use the ALI standard, compared to those that use the M’Naghten [2]. In addition, the role of inhibitory control and executive functions is supported by neuroscientific studies, showing the presence of cerebral alterations in violent offenders, specifically prefrontal dysfunctions [32–39].

The absence of significant differences between final judgements of forensic cases evaluated with and without the use of the DIASS, suggests that our instrument does not alter the decisional process typically used in forensic experts’ practice, despite improving the transparency and verifiability of judgment. In this regard, it is worth underlining that the scale may be apparently of little use for those forensic experts with many years of experience. However, we deem that the DIASS could be useful also for experienced forensic experts, as it would allow them to make explicit in the forensic reports the patterns of reasoning that are generally implicit, making them more objective. In addition, the DIASS could be especially useful to those who are at the beginning of the forensic profession as it could act as support and guidance for the evaluation of the defendant’s accountability as well as the training of forensic specialists.

From the analysis of the correlations between the defendants’ capacities analyzed through the DIASS and the psychopathological dimension measured by the BPRS, three different profiles of forensic experts’ decision-making emerged. While regarding defendants judged nonresponsible the impairment of the capacities analyzed through the items of the DIASS (such as the ability to control one’s own behavior, etc.) were mainly associated with the mania/excitement dimension, for defendants with substantially diminished responsibility the most affected psychopathological dimension was represented by positive symptoms on the BPRS. This is in line with some studies reporting positive, negative, and manic symptoms as to be more frequently associated with judgments of nonresponsibility and could be considered as generally related to the impact of the “psychotic dimension” on responsibility [25, 40, 41]. Finally, regarding responsible defendants, the psychopathological dimensions most involved in the impairment of the cognitive-volitional components were those of hostility and negative symptoms, probably reflecting the fact that our responsible sample was mainly composed of defendants affected by personality disorders such as antisocial, borderline or paranoid, with high levels of anger and hostility. Differently from defendants judged nonresponsible and with substantially diminished responsibility, where the presence of psychotic symptoms plays a role in forensic experts’ decisional processes, regarding responsible defendants, the psychiatric dimension of hostility and negative symptoms (composed of motor retardation, blunted affect, disorientation, and self-neglect) are not associated with a judgement of nonresponsibility, but merely present. In interpreting these results, we must consider that these correlations refer to the psychopathological dimensions present in our forensic hypothetical case (based on real forensic cases modified to make them not recognizable) and that the BPRS referred to the state of mind at the time of the forensic evaluation, as it has been reported in the forensic report, and not at the time of the crime. A possible limitation of this study is represented by the limited number and typology of hypothetical forensic cases (although based on real cases modified to make them unrecognizable) and the reduced number of forensic experts (*N* = 40) who evaluated the cases.

Despite the DIASS having been validated on forensic cases based on the Italian penal code, it can also be used in those countries where insanity criteria follow the M’Naghten Rule or the ALI standard. In such cases, the evaluators should focus more on those components of the DIASS that refer to the criterion deemed most relevant within a specific jurisdiction. Furthermore, it could be useful in those states such as the Netherlands where no specific legal standards have been formulated to establish when a person is criminally responsible for their actions. In addition, there is a need for cross-cultural validation within both continental European and Common Law legislation.

Moreover, it represents the first step toward a reduction of the heterogeneity of forensic evaluations and toward standardization of their methodology, thus leading to an improvement in the whole criminal justice process.

## Supplementary information

Supplementary data file

Appendix A

## Footnotes

1 M’Naghten’s Case, 10 Cl. & Fin. 200, 8 Eng. Rep. 718 (H.L. 1843

2 Articles 88 and 89 of the Italian Penal Code establish the criteria for determining whether an offender can be considered nonresponsible o with substantially diminished responsibility, as in the following: “a person who, at the moment in which he/she committed the crime, was, because of infirmity, in such a state of mind as to exclude the capacity of appreciating or willing, is nonresponsible” (Article 88); “a person who, at the moment in which he/she committed the crime, was, because of infirmity, in such a state of mind as to greatly diminish, without excluding, the capacity of appreciating and willing, is responsible for the crime committed, but the penalty is reduced” (Article 89)
