# Supplementary material for: Validation of a new instrument to guide and support insanity evaluations: the defendant’s insanity assessment support scale (DIASS)
Source: Transl Psychiatry. 2022 Mar 22;12:115. doi: 10.1038/s41398-022-01871-8 (PMC8941181; doi:10.1038/s41398-022-01871-8)
Supplement: Supplementary file 1 — Supplementary data file [file 41398_2022_1871_MOESM1_ESM.docx]

**FORENSIC CASE N1 - Schizophrenia**

**The crime**

The defendant is a 40-year-old man, in prison for killing his father and hitting his brother, causing him “contusive polytrauma, multiple lacerated-contused wounds, and a minor head injury”.

**Anamnesis**

The mother died of breast cancer at the age of 58, she was a housewife.

The father died at the age of 68, due to posttraumatic cerebral haemorrhaging and post-traumatic respiratory failure reported following the injuries referred to in the crimes in question. He worked as a plumber.

He is the second of two children: he has a 47-year-old brother with congenital severe intellectual disability who has always lived with his brother and father.

A poor academic performance is reported (he failed second year of middle school), he obtained a lower middle school diploma and then worked as a plumber and as a dog-sitter. He describes himself as introverted, he never had a romantic relationship. He performed military service. He does not take alcohol or drugs; he smokes about 20 cigarettes a day.

A first contact with the local mental health center is reported when the defendant was 25 years old, because of persecutory delusional ideation and imperative hallucinations. On that occasion, a pharmacotherapy based on mood stabilizers and antipsychotics was then set up, autonomously suspended by the defendant because he was "well".

When the defendant was 35 years old, he was admitted to a psychiatric ward because of persecutory delusion and imperative hallucinations. Since discharge he was taken in charge by the mental health center with depot antipsychotic therapy. No previous aggressive acts are described, neither hetero nor self-directed, nor previous problems with justice.

On the morning of the crime, the defendant goes, as always, to the family where he works as dog-sitter. In the afternoon, the defendant contacts his psychiatrist and claims to have killed his father. The day care center operators then go to the defendant’s home, and on their arrival, he declares that the father "was sick". The operators, having ascertained the situation, call the emergency number (118).

In the days following the crime the defendant appears to be inaccessible for interview, mutacic and scarcely aware of what happened.

**Status**

At the forensic psychiatric evaluation, he is poorly accessible for the interview. He appears poorly taken care of in appearance and personal hygiene. The mimicry is hypomobile, the motility is slowed. The speech, not spontaneous, needs to be elicited with questions posed by the examiner.

He claims to know that he is confined in jail for assaulting his father and brother but he sustains he does not remember anything about it. He claims to be aware of the death of his father with poor emotional resonance.

During the interview, he appears to be distracted by internal and external stimuli with slowed thinking and long-lasting thought blocking. While not remembering anything of the aggression, he reports that at the exit from the home of the family where he works as a dog-sitter, while he was on the street, he could have experienced a progressive feeling that the world around him had become "different, empty, there was nothing", probable a phenomenon of delusional perception, then he claims not to remember anything else about that day.

Invited to describe if he had ever had the feeling that his own thought was controlled from outside, he stated, with a certain latency, “it is possible”, and he admitted to have heard a voice that “he does not want me to say that I am controlled”. He also described having in the past had psychopathological phenomena attributable to delusions of thought insertion and thought control as well as reference and persecutory delusions and imperative hallucinations.

# BRIEF PSYCHIATRIC RATING SCALE (BPRS) (version 4.0)

# Scoring

*Evaluate items 1-14 on the basis of what the patient says during the interview. Mark "NA" for symptoms that have not been evaluated. Note: items 7, 12 and 13 must be also coded on the basis of the behavior observed during the interview. Remember that the scale to be used is the following*

| **NA** | **1** | **2** | **3** | **4** | **5** | **6** | **7** |
| --- | --- | --- | --- | --- | --- | --- | --- |
| not assessed | not present | very mild | mild | moderate | moderately severe | severe | extremely severe |

| 1. Somatic concern | NV | 1 | 2 | 3 | 4 | 5 | 6 | 7 |  |
| --- | --- | --- | --- | --- | --- | --- | --- | --- | --- |
| 2. Anxiety | NV | 1 | 2 | 3 | 4 | 5 | 6 | 7 |  |
| 3. Depression | NV | 1 | 2 | 3 | 4 | 5 | 6 | 7 |  |
| 4. Suicidality | NV | 1 | 2 | 3 | 4 | 5 | 6 | 7 |  |
| 5. Guilt | NV | 1 | 2 | 3 | 4 | 5 | 6 | 7 |  |
| 6. Hostility | NV | 1 | 2 | 3 | 4 | 5 | 6 | 7 |  |
| 7. Elevated Mood | NV | 1 | 2 | 3 | 4 | 5 | 6 | 7 |  |
| 8. Grandiosity | NV | 1 | 2 | 3 | 4 | 5 | 6 | 7 |  |
| 9. Suspiciousness | NV | 1 | 2 | 3 | 4 | 5 | 6 | 7 |  |
| 10. Hallucinations | NV | 1 | 2 | 3 | 4 | 5 | 6 | 7 |  |
| 11. Unusual thoughts content | NV | 1 | 2 | 3 | 4 | 5 | 6 | 7 |  |
| 12. Bizarre behavior | NV | 1 | 2 | 3 | 4 | 5 | 6 | 7 |  |
| 13. Self-neglect | NV | 1 | 2 | 3 | 4 | 5 | 6 | 7 |  |
| 14. Disorientation | NV | 1 | 2 | 3 | 4 | 5 | 6 | 7 |  |

*Evaluate the following items 15 to 24 on the basis of the patient's speech and behavior during the interview.*

| 15. Conceptual disorganization | NV | 1 | 2 | 3 | 4 | 5 | 6 | 7 |  |
| --- | --- | --- | --- | --- | --- | --- | --- | --- | --- |
| 16. Blunted affect | NV | 1 | 2 | 3 | 4 | 5 | 6 | 7 |  |
| 17. Emotional withdrawal | NV | 1 | 2 | 3 | 4 | 5 | 6 | 7 |  |
| 18. Motor retardation | NV | 1 | 2 | 3 | 4 | 5 | 6 | 7 |  |
| 19. Tension | NV | 1 | 2 | 3 | 4 | 5 | 6 | 7 |  |
| 20. Uncooperativeness | NV | 1 | 2 | 3 | 4 | 5 | 6 | 7 |  |
| 21. Excitement | NV | 1 | 2 | 3 | 4 | 5 | 6 | 7 |  |
| 22. Distractibility | NV | 1 | 2 | 3 | 4 | 5 | 6 | 7 |  |
| 23. Motor hyperactivity | NV | 1 | 2 | 3 | 4 | 5 | 6 | 7 |  |
| 24. Mannerisms and posturing | NV | 1 | 2 | 3 | 4 | 5 | 6 | 7 |  |

**FORENSIC CASE N2 – Bipolar Disorder**

**The crime**

The defendant is a 28-year-old man, in prison because “with repeated conduct harassed and threatened S. A. (aged 23), not accepting the end of their relationship. He went several times to the victim's home asking her to resume their relationship, telephoning her and texting her insistently and threatening her mother with death, because, in his opinion, she was against the resumption of their romantic relationship. Consequently, the victim, fearing for her own safety, changed her life habits, changing her phone number and avoiding leaving the house in order not to meet him.

**Anamnesis**

The mother is 54 years-old and she is a teacher. Their relationship is described as conflictual.

The father is 58-year-old, he is a clerk, and he is in apparent good health.

He is an only child. His parents split up when the defendant was 6 years old. The maternal grandmother is affected by bipolar disorder.

The defendant describes himself as a "very cheerful, lively” child. A poor academic performance is reported (he failed first year of middle school, a suspension for disciplinary reasons is reported). Subsequently he attended, for about 2 years, a tourism college, before quitting his studies to devote himself to the practice of karate. In this regard, discrete results are reported with fluctuating successes, both because of his mental health condition and the use of drugs. He refers to have occasionally worked as a "bouncer", but he never had an employment contract. He reports intake of cannabinoids from the age of about 15, and cocaine for sniffing at the age of about 17. He refers to sporadically abusing spirits, denying a persistent problem in this regard.

The onset of psychiatric conditions dates back to the age of 20, apparently following the intake of cocaine, which would have resulted in the development of persecutory delusional ideation with auditory and visual hallucinations. Since then, he was taken in charge by the local Drug Rehabilitation Service, with alternating periods of clinical compensation and taking therapy, and relapses often associated with the use of substances.

When the defendant was 27 years old, he was involuntarily committed in an acute psychiatric ward, for a psychotic episode. The toxicological test was negative for cocaine. As a stressful factor associated with the relapse, the end of the romantic relationship with the aforementioned S. A was reported. The defendant presented a delusion focused on the belief that the girl was still in love with him and that she left him at the insistence of the mother who did not approve of their relationship; the presence of this idea at that time was also confirmed by the defendant, with minimizing methods. After discharge he was transferred to a psychiatric clinic, were he stayed for about 20 days and received a diagnosis of Bipolar Disorder, type I. The continuation of treatment was attempted at a Therapeutic Community, which he left after about 10 days. Subsequent attempts to reintegrate him proved unsuccessful due to the refusal of the defendant. There was also discontinuity in attending the Drug Rehabilitation Service. Two months ago, he was again involuntary committed in an acute psychiatric ward, and was affected by "delusional state"; he reported having taken cocaine a few days before, at the interview contents of delusional thought emerged with a megalomaniac background.

**Status**

The defendant is quite smart in appearance and personal hygiene, he has an athletic build. He shows a minimizing attitude both towards the abusive conduct of drugs and towards the manifested psychopathological symptoms; a similar attitude has been shown in relation to his own legal proceedings. He is alert and oriented in the three axes.

He admitted that he went several times to the home of his ex-girlfriend, S. A., because he was convinced that the girl was still in love with him and that she left him at the insistence of the mother who did not approve of their relationship, despite having currently admitted that he could not refer to real elements supporting this hypothesis.

Attention did not show clinically detectable alterations during the Psychiatric interview. Absent formal-logical alterations of thought. However, there is some ideational poverty, combined with a fairly basic suspicion and a tendency to interpretativeness. From the analysis of the delusional ideation that he presented in the past, as well as serious behavioral alterations, however, no critical ability emerged. Feelings of anxiety also emerged, with concern for the outcome of the trial. The mood did not show depressive or excitative aspects, however a marked lability is noticeable, as well as the presence of some notes of dysphoria, tending to be minimized by the defendant. Affectivity has been characterized by the absence of flattening, however fatuity and childish traits are appreciated. Impulse control is structurally problematic. There has been no appreciation of hetero or self-injurious ideas. There are no signs that suggests the presence of hallucinations, nor did the subject report them.

Disease awareness is marginal, as is the ability to understand and adequately adhere to a care project.

# BRIEF PSYCHIATRIC RATING SCALE (BPRS) (version 4.0)

# Scoring

*Evaluate items 1-14 on the basis of what the patient says during the interview. Mark "NA" for symptoms that have not been evaluated. Note: items 7, 12 and 13 must be also coded on the basis of the behavior observed during the interview. Remember that the scale to be used is the following*

| **NA** | **1** | **2** | **3** | **4** | **5** | **6** | **7** |
| --- | --- | --- | --- | --- | --- | --- | --- |
| not assessed | not present | very mild | mild | moderate | moderately severe | severe | extremely severe |

| 1. Somatic concern | NV | 1 | 2 | 3 | 4 | 5 | 6 | 7 |  |
| --- | --- | --- | --- | --- | --- | --- | --- | --- | --- |
| 2. Anxiety | NV | 1 | 2 | 3 | 4 | 5 | 6 | 7 |  |
| 3. Depression | NV | 1 | 2 | 3 | 4 | 5 | 6 | 7 |  |
| 4. Suicidality | NV | 1 | 2 | 3 | 4 | 5 | 6 | 7 |  |
| 5. Guilt | NV | 1 | 2 | 3 | 4 | 5 | 6 | 7 |  |
| 6. Hostility | NV | 1 | 2 | 3 | 4 | 5 | 6 | 7 |  |
| 7. Elevated Mood | NV | 1 | 2 | 3 | 4 | 5 | 6 | 7 |  |
| 8. Grandiosity | NV | 1 | 2 | 3 | 4 | 5 | 6 | 7 |  |
| 9. Suspiciousness | NV | 1 | 2 | 3 | 4 | 5 | 6 | 7 |  |
| 10. Hallucinations | NV | 1 | 2 | 3 | 4 | 5 | 6 | 7 |  |
| 11. Unusual thoughts content | NV | 1 | 2 | 3 | 4 | 5 | 6 | 7 |  |
| 12. Bizarre behavior | NV | 1 | 2 | 3 | 4 | 5 | 6 | 7 |  |
| 13. Self-neglect | NV | 1 | 2 | 3 | 4 | 5 | 6 | 7 |  |
| 14. Disorientation | NV | 1 | 2 | 3 | 4 | 5 | 6 | 7 |  |

*Evaluate the following items 15 to 24 on the basis of the patient's speech and behavior during the interview.*

| 15. Conceptual disorganization | NV | 1 | 2 | 3 | 4 | 5 | 6 | 7 |  |
| --- | --- | --- | --- | --- | --- | --- | --- | --- | --- |
| 16. Blunted affect | NV | 1 | 2 | 3 | 4 | 5 | 6 | 7 |  |
| 17. Emotional withdrawal | NV | 1 | 2 | 3 | 4 | 5 | 6 | 7 |  |
| 18. Motor retardation | NV | 1 | 2 | 3 | 4 | 5 | 6 | 7 |  |
| 19. Tension | NV | 1 | 2 | 3 | 4 | 5 | 6 | 7 |  |
| 20. Uncooperativeness | NV | 1 | 2 | 3 | 4 | 5 | 6 | 7 |  |
| 21. Excitement | NV | 1 | 2 | 3 | 4 | 5 | 6 | 7 |  |
| 22. Distractibility | NV | 1 | 2 | 3 | 4 | 5 | 6 | 7 |  |
| 23. Motor hyperactivity | NV | 1 | 2 | 3 | 4 | 5 | 6 | 7 |  |
| 24. Mannerisms and posturing | NV | 1 | 2 | 3 | 4 | 5 | 6 | 7 |  |

**FORENSIC CASE N3 – Delusional Disorder, mixed type, continuous**

**The crime**

The defendant is a 45-year-old man, in prison for attempting to poison his wife and for repeated abuse of his wife through beatings and insults perpetrated over the past 4 years.

**Anamnesis**

His father died at the age of 65 from myocardial infarction.

The mother is 71 years old and she is affected by type II diabetes.

He is the second of 4 children: he has a 48-year-old brother, affected by alcoholic abuse, a 43-year-old sister, who is disabled from birth, and a 40-year-old sister suffering from an unspecified psychiatric disease.

He has a high school diploma. He then carried out several jobs, including bricklayer and truck driver. For several years, he carried out occasional jobs.

At the age of 28 he married a woman 5 years younger than him.

In 2017 he was accompanied by his wife to the mental health center for reasons that he does not know and begins antipsychotic therapy (oral and intramuscular), interrupted autonomously after about 1 year.

He says that since 2017 he noticed that his wife was cheating on him with many men through pornographic films found on the internet, having recognized her by the physique and how she spoke and he was also convinced that his wife was prostituting herself in the street. When he confronted his wife on the matter, she reacted angrily, calling him "crazy" and accompanying him to the mental health center. Regarding to the aggressive conduct towards his wife, which the defendant admits, he declares that his wife wanted to kill him.

**Status**

At the forensic psychiatric evaluation, he is quite smart in appearance and personal hygiene. He is alert and presents a good orientation over time, space and in relation to the context. Eye contact is maintained throughout the interview, with the exception of the moments when the defendant speaks of the wife. A moderate level of tension in the body is appreciated.

The speech is fluid, the tone of voice modulated.

A delusional ideation emerges with contents of jealousy and of persecutory type towards his wife.

He is convinced that his wife has betrayed him with many men and he claims he found evidence of this in pornographic films that he allegedly found on the internet, however he is unable to bring details that can rationally link the films to his wife. Persecutory ideas also emerge, always towards his wife, with the belief that she wanted to kill him in the past, also through the administration of medicines, in particular psychotropic drugs and food.

He also reports the previous delusional belief that his wife had induced him erectile dysfunction through the administration of medicine. A slight loosening of the associative connections is appreciated. Alterations in the senses of perception do not emerge, nor does the defendant report them. The mood appears dysphoric, there is a moderate level of anxiety. Cognitive functioning appears to be free of alterations detectable at the interview.

# BRIEF PSYCHIATRIC RATING SCALE (BPRS) (version 4.0)

# Scoring

*Evaluate items 1-14 on the basis of what the patient says during the interview. Mark "NA" for symptoms that have not been evaluated. Note: items 7, 12 and 13 must be also coded on the basis of the behavior observed during the interview. Remember that the scale to be used is the following*

| **NA** | **1** | **2** | **3** | **4** | **5** | **6** | **7** |
| --- | --- | --- | --- | --- | --- | --- | --- |
| not assessed | not present | very mild | mild | moderate | moderately severe | severe | extremely severe |

| 1. Somatic concern | NV | 1 | 2 | 3 | 4 | 5 | 6 | 7 |  |
| --- | --- | --- | --- | --- | --- | --- | --- | --- | --- |
| 2. Anxiety | NV | 1 | 2 | 3 | 4 | 5 | 6 | 7 |  |
| 3. Depression | NV | 1 | 2 | 3 | 4 | 5 | 6 | 7 |  |
| 4. Suicidality | NV | 1 | 2 | 3 | 4 | 5 | 6 | 7 |  |
| 5. Guilt | NV | 1 | 2 | 3 | 4 | 5 | 6 | 7 |  |
| 6. Hostility | NV | 1 | 2 | 3 | 4 | 5 | 6 | 7 |  |
| 7. Elevated Mood | NV | 1 | 2 | 3 | 4 | 5 | 6 | 7 |  |
| 8. Grandiosity | NV | 1 | 2 | 3 | 4 | 5 | 6 | 7 |  |
| 9. Suspiciousness | NV | 1 | 2 | 3 | 4 | 5 | 6 | 7 |  |
| 10. Hallucinations | NV | 1 | 2 | 3 | 4 | 5 | 6 | 7 |  |
| 11. Unusual thoughts content | NV | 1 | 2 | 3 | 4 | 5 | 6 | 7 |  |
| 12. Bizarre behavior | NV | 1 | 2 | 3 | 4 | 5 | 6 | 7 |  |
| 13. Self-neglect | NV | 1 | 2 | 3 | 4 | 5 | 6 | 7 |  |
| 14. Disorientation | NV | 1 | 2 | 3 | 4 | 5 | 6 | 7 |  |

*Evaluate the following items 15 to 24 on the basis of the patient's speech and behavior during the interview.*

| 15. Conceptual disorganization | NV | 1 | 2 | 3 | 4 | 5 | 6 | 7 |  |
| --- | --- | --- | --- | --- | --- | --- | --- | --- | --- |
| 16. Blunted affect | NV | 1 | 2 | 3 | 4 | 5 | 6 | 7 |  |
| 17. Emotional withdrawal | NV | 1 | 2 | 3 | 4 | 5 | 6 | 7 |  |
| 18. Motor retardation | NV | 1 | 2 | 3 | 4 | 5 | 6 | 7 |  |
| 19. Tension | NV | 1 | 2 | 3 | 4 | 5 | 6 | 7 |  |
| 20. Uncooperativeness | NV | 1 | 2 | 3 | 4 | 5 | 6 | 7 |  |
| 21. Excitement | NV | 1 | 2 | 3 | 4 | 5 | 6 | 7 |  |
| 22. Distractibility | NV | 1 | 2 | 3 | 4 | 5 | 6 | 7 |  |
| 23. Motor hyperactivity | NV | 1 | 2 | 3 | 4 | 5 | 6 | 7 |  |
| 24. Mannerisms and posturing | NV | 1 | 2 | 3 | 4 | 5 | 6 | 7 |  |

**FORENSIC CASE N 4 – Delusional disorder/Schizophrenia**

**The crime**

The defendant is a 32-year-old man, accused of having poisoned with arsenic his father, and his brother.

From the investigations carried out, it emerged that the subject had managed to obtain the arsenic, making repeated online purchases. The computer investigations have highlighted how the subject used a fictitious email address and name for this purpose, created ad hoc for the purchase of the chemical compound. It was also highlighted by the analysis of the computers used that part of the related traces (History and cache) had been deleted. The defendant had also collected the material personally in Florence, always paying in cash.

**Anamnesis**

The father, poisoned by the defendant, was 60 years old and was a plumber. The mother, who is 60 years old, is a teacher, and suffered from depression some years ago. He is the first of two children: the brother, who was 22 years old was poisoned by the defendant.

Apart from the mother, there is no family history of psychiatric or neurological diseases.

He finished high school, but repeated the first year. He did military service, after which he performed different jobs, such as waiter and shop assistant until 2015, since then he has been unemployed. He reports alcohol abuse (7-8 beers per day), apparently in a phase characterized by work problems, and stop taking alcohol 2 years ago. He never had a romantic relationship, nor does he claim to have had sex.

He reports a first contact with a psychologist at the age of 12, perhaps due to relationship problems.

He refers, during adolescence, the presence of mood deflection, because of difficulties in school, which he does not know how to specify better. He denies other psychiatric disorders, despite acknowledging the current presence of thymic deflection and insomnia.

The defendant’s mother reported that during the last two years her son had become introverted and had limited relationships with them. The defendant used to spend his days locked up in his room. He had stopped driving because he couldn't stand having cars near him, and he no longer used public transport. She referred that in the last period her son had lost all of his friends and that he went for a period to a psychologist, with little benefit. In the last 2 years he imposed on himself a lifestyle characterized by limited nutrition, removing every ornament from the room and eliminating sweets and alcohol.

**Status**

At the forensic psychiatric evaluation, the defendant is poorly cared for in appearance and personal hygiene; he is alert, oriented in time, space and in relation to the context.

Despite a courteous and formally collaborative approach, an evident difficulty in showing empathy both with respect to the interlocutors and to the victims of his conduct emerges.

Facial mimicry is considerably reduced, gloomy, marked by a severe and oppressed attitude. He stares back at the evaluator just for brief moments, and presents a poorly represented non-verbal communication, marked by a condition of apathy.

He speaks with fluency, yet sometimes with some latencies and with the use of archaic sentences, almost of biblical inspiration.

A delusional ideation with mystic-religious content emerges. About three years ago the "discrepancies" started with his parents and his brother, because of the latter behaving in a way he did not approve, such as tv programs they watched and the way they dressed. According to the defendant “these things” (behavior of his family) were wrong and “he wanted them to stop acting that way, through their death, because this was the only way”. He therefore poisoned the water of the family members with arsenic. He adds that now he has repented.

He denies the presence of hallucinations, despite having referred to their presence in the period preceding the crime (“the voice of God calling me to him”). The content of the hallucinations seems to conform to the thought disorder.

The mood is in line, although nuanced notes of demoralization are appreciated. Affectivity is constricted. The ability to carry out symbolic and abstract thinking appears to be significantly reduced. Awareness of illness is limited.

CAT scan with negative result

# BRIEF PSYCHIATRIC RATING SCALE (BPRS) (version 4.0)

# Scoring

*Evaluate items 1-14 on the basis of what the patient says during the interview. Mark "NA" for symptoms that have not been evaluated. Note: items 7, 12 and 13 must be also coded on the basis of the behavior observed during the interview. Remember that the scale to be used is the following*

| **NA** | **1** | **2** | **3** | **4** | **5** | **6** | **7** |
| --- | --- | --- | --- | --- | --- | --- | --- |
| not assessed | not present | very mild | mild | moderate | moderately severe | severe | extremely severe |

| 1. Somatic concern | NV | 1 | 2 | 3 | 4 | 5 | 6 | 7 |  |
| --- | --- | --- | --- | --- | --- | --- | --- | --- | --- |
| 2. Anxiety | NV | 1 | 2 | 3 | 4 | 5 | 6 | 7 |  |
| 3. Depression | NV | 1 | 2 | 3 | 4 | 5 | 6 | 7 |  |
| 4. Suicidality | NV | 1 | 2 | 3 | 4 | 5 | 6 | 7 |  |
| 5. Guilt | NV | 1 | 2 | 3 | 4 | 5 | 6 | 7 |  |
| 6. Hostility | NV | 1 | 2 | 3 | 4 | 5 | 6 | 7 |  |
| 7. Elevated Mood | NV | 1 | 2 | 3 | 4 | 5 | 6 | 7 |  |
| 8. Grandiosity | NV | 1 | 2 | 3 | 4 | 5 | 6 | 7 |  |
| 9. Suspiciousness | NV | 1 | 2 | 3 | 4 | 5 | 6 | 7 |  |
| 10. Hallucinations | NV | 1 | 2 | 3 | 4 | 5 | 6 | 7 |  |
| 11. Unusual thoughts content | NV | 1 | 2 | 3 | 4 | 5 | 6 | 7 |  |
| 12. Bizarre behavior | NV | 1 | 2 | 3 | 4 | 5 | 6 | 7 |  |
| 13. Self-neglect | NV | 1 | 2 | 3 | 4 | 5 | 6 | 7 |  |
| 14. Disorientation | NV | 1 | 2 | 3 | 4 | 5 | 6 | 7 |  |

*Evaluate the following items 15 to 24 on the basis of the patient's speech and behavior during the interview.*

| 15. Conceptual disorganization | NV | 1 | 2 | 3 | 4 | 5 | 6 | 7 |  |
| --- | --- | --- | --- | --- | --- | --- | --- | --- | --- |
| 16. Blunted affect | NV | 1 | 2 | 3 | 4 | 5 | 6 | 7 |  |
| 17. Emotional withdrawal | NV | 1 | 2 | 3 | 4 | 5 | 6 | 7 |  |
| 18. Motor retardation | NV | 1 | 2 | 3 | 4 | 5 | 6 | 7 |  |
| 19. Tension | NV | 1 | 2 | 3 | 4 | 5 | 6 | 7 |  |
| 20. Uncooperativeness | NV | 1 | 2 | 3 | 4 | 5 | 6 | 7 |  |
| 21. Excitement | NV | 1 | 2 | 3 | 4 | 5 | 6 | 7 |  |
| 22. Distractibility | NV | 1 | 2 | 3 | 4 | 5 | 6 | 7 |  |
| 23. Motor hyperactivity | NV | 1 | 2 | 3 | 4 | 5 | 6 | 7 |  |
| 24. Mannerisms and posturing | NV | 1 | 2 | 3 | 4 | 5 | 6 | 7 |  |

**FORENSIC CASE N 5 – Schizoaffective disorder**

**The crime**

The defendant is a 39-year-old woman, who during her stay at a Drug Rehabilitation Community, murdered her 3-month-old son.

**Anamnesis**

The mother is 59 years-old, she is a housewife. She separated from the defendant's father when the daughter was 5 years old. Over the years she suffered from postpartum psychosis and several depressive relapses.

The father is 61 years old and he is engineer. He never took care of the mother or daughter. He was treated for problems related to alcohol abuse.

Psychiatric familiarity: in addition to the mother, the paternal grandmother was affected by schizophrenia.

Due to the frequent hospitalizations of the mother, the defendant was often entrusted to maternal grandparents.

She reports that she was a very shy child and had always had difficulty relating to peers. A first psychiatric hospitalization is reported at the age of 20 for an attempted suicide (incongruous ingestion of drugs); on that occasion a "borderline personality disorder" was diagnosed. Then followed by another 3 psychiatric hospitalizations from which she is discharged with the diagnosis of "schizoaffective disorder with substance and alcohol abuse" and she is treated with therapies based on antipsychotics, mood stabilizers and benzodiazepines. Schooling: high school diploma. She worked as shop assistant for a certain period.

She has had several conflicting sentimental relationships. At the age of 36 she met her current partner, from whom she had a son. She refers to previous alcohol and substance abuse, interrupted for the pregnancy and resumed in the month preceding the crime (which occurred about 24 days after her entry into Drug Rehabilitation Community. During this period, laboratory data show that the defendant was clean).

**Status**

At the forensic psychiatric evaluation, the defendant appears poorly cared for in appearance and personal hygiene. She is oriented in time, space and in relation to the context. She accesses the interview in an available and collaborative way, despite showing some discomfort.

The mimicry is marked by sadness, motor skills appear slowed down, but not psychism; the speech, at times, is accelerated.

Regarding the crime, she reports that as soon as she entered the Drug Rehabilitation Community, she immediately began to fear for the safety of his son (listening to pieces of speeches from other patients in the community she understood that satanic masses were made in that place with human sacrifices, especially children). She felt that nobody could be trusted, that everyone was part of a plot to take her son away to harm him. She began to interpret some external signals in a self-referenced sense (e.g. a woman with a red shirt, noises from outside), developing the belief that it was the signal of an imminent kidnapping of her son, who would then be tortured, killed and offered for sacrifice. Therefore, one morning she took her son, went away with him and threw him upside down from the window; she was convinced that "sending him to heaven would save him."

At the psychiatric evaluation which followed immediately after the crime, given the seriousness of the psychiatric symptomatology found and in the presence of a potential risk of self-injury, the patient was transferred to an acute psychiatric ward on an involuntary hospitalization basis.

The defendant shows an initial criticism of the gesture which is not explicitly criticized for her purpose of "saving his son". However, she appears detached from the action taken. Absent perceptual disturbances.

The affectivity is constrained, the emotionality is labile. The mood is deflected, significant levels of anxiety are not appreciated.

# BRIEF PSYCHIATRIC RATING SCALE (BPRS) (version 4.0)

# Scoring

*Evaluate items 1-14 on the basis of what the patient says during the interview. Mark "NA" for symptoms that have not been evaluated. Note: items 7, 12 and 13 must be also coded on the basis of the behavior observed during the interview. Remember that the scale to be used is the following*

| **NA** | **1** | **2** | **3** | **4** | **5** | **6** | **7** |
| --- | --- | --- | --- | --- | --- | --- | --- |
| not assessed | not present | very mild | mild | moderate | moderately severe | severe | extremely severe |

| 1. Somatic concern | NV | 1 | 2 | 3 | 4 | 5 | 6 | 7 |  |
| --- | --- | --- | --- | --- | --- | --- | --- | --- | --- |
| 2. Anxiety | NV | 1 | 2 | 3 | 4 | 5 | 6 | 7 |  |
| 3. Depression | NV | 1 | 2 | 3 | 4 | 5 | 6 | 7 |  |
| 4. Suicidality | NV | 1 | 2 | 3 | 4 | 5 | 6 | 7 |  |
| 5. Guilt | NV | 1 | 2 | 3 | 4 | 5 | 6 | 7 |  |
| 6. Hostility | NV | 1 | 2 | 3 | 4 | 5 | 6 | 7 |  |
| 7. Elevated Mood | NV | 1 | 2 | 3 | 4 | 5 | 6 | 7 |  |
| 8. Grandiosity | NV | 1 | 2 | 3 | 4 | 5 | 6 | 7 |  |
| 9. Suspiciousness | NV | 1 | 2 | 3 | 4 | 5 | 6 | 7 |  |
| 10. Hallucinations | NV | 1 | 2 | 3 | 4 | 5 | 6 | 7 |  |
| 11. Unusual thoughts content | NV | 1 | 2 | 3 | 4 | 5 | 6 | 7 |  |
| 12. Bizarre behavior | NV | 1 | 2 | 3 | 4 | 5 | 6 | 7 |  |
| 13. Self-neglect | NV | 1 | 2 | 3 | 4 | 5 | 6 | 7 |  |
| 14. Disorientation | NV | 1 | 2 | 3 | 4 | 5 | 6 | 7 |  |

*Evaluate the following items 15 to 24 on the basis of the patient's speech and behavior during the interview.*

| 15. Conceptual disorganization | NV | 1 | 2 | 3 | 4 | 5 | 6 | 7 |  |
| --- | --- | --- | --- | --- | --- | --- | --- | --- | --- |
| 16. Blunted affect | NV | 1 | 2 | 3 | 4 | 5 | 6 | 7 |  |
| 17. Emotional withdrawal | NV | 1 | 2 | 3 | 4 | 5 | 6 | 7 |  |
| 18. Motor retardation | NV | 1 | 2 | 3 | 4 | 5 | 6 | 7 |  |
| 19. Tension | NV | 1 | 2 | 3 | 4 | 5 | 6 | 7 |  |
| 20. Uncooperativeness | NV | 1 | 2 | 3 | 4 | 5 | 6 | 7 |  |
| 21. Excitement | NV | 1 | 2 | 3 | 4 | 5 | 6 | 7 |  |
| 22. Distractibility | NV | 1 | 2 | 3 | 4 | 5 | 6 | 7 |  |
| 23. Motor hyperactivity | NV | 1 | 2 | 3 | 4 | 5 | 6 | 7 |  |
| 24. Mannerisms and posturing | NV | 1 | 2 | 3 | 4 | 5 | 6 | 7 |  |

**FORENSIC CASE N6 – Other Specified Personality Disorder, mixed personality features (Histrionic/Narcisistic)**

**The crime**

The defendant is a 45-year-old woman, accused of attempted murder of her friend C. D., for hitting the victim violently on the head in the front-temporal area twice with a wrench. The victim initially reacted by screaming and partially blocking the first blow of the hammer with her hands, thus mitigating the impact, and then, after the second blow, she fell to the ground pretending to be lifeless. The injuries reported consisted of "commotional head injury, loss of urine, right front-temporal contusion lacerated wound". The defendant had previously threatened the victim and her children with death because, according to her, she emitted a negative energy that damaged and disturbed her.

**Anamnesis**

The mother is 75 years old, she is retired, she used to work as a clerk.

The father is 80 years old, he is retired, he used to work as a clerk.

She is the first of two children: the sister is 42 years old and is in apparent good health.

She denies psychiatric and neurological familiarity

She has a degree in Literature even though she has always worked in the field of fashion and event organization.

She reports a series of conflictual sentimental relationships. She married at the age of 30 a man with whom she had a 13-year-old son who lives with his maternal grandparents. She separated from this man when her son was 6 years old. She refers that, although the son does not live with her, because for work reasons she had had to move to a different city, the defendant sees him on a regular basis.

**Status**

At the forensic psychiatric evaluation, she appears smart in appearance and personal hygiene. She is oriented in time, space and in relation to the context.

She has an adequate and formally available approach, with manipulative and seductive attitudes, sometimes tending to dramatization. Mimicry is mobile, marked by sadness and tears, but modulated with respect to the issues addressed. The gaze supports that of the examiners, the speech is fluid and spontaneous.

There are no frank disturbances in the form or content of the thought, although the patient appears at times to be vaguely interpretative. The contents of the thought focus on concerns about her future, on experiences of greatness (she claims to be an artist and brags important acquaintanceships and friendships), and on issues related to the reasons why she is currently in prison. There are no direct and / or indirect signs of perceptive disturbances, although the defendant reports the presence of auditory hallucinations, described vaguely and reported without any emotional participation.

The mood is oriented in a depressive sense, affectivity is labile. There is a mild level of free anxiety and concerns about her future. Cognitive functions appear well preserved, consistent in relation to age and schooling. Regarding the crime, the defendant reports that she has known the victim for about 10 years, to have been close to her when the latter lost her husband. The defendant reports the breakdown of this friendship following the victim's sentimental interest in a man with whom the defendant had had a previous relationship (ended at the behest of the defendant). She also reports that that morning she had gone to the victim's private practice at the closing time with the intention of a clarification, but that the victim had taken her by the hair by starting a fight during which a hammer came out of the defendant's bag (which she claims to always carry with her for personal defense) that incidentally hit the victim.

She also claims to have moved away from the victim's private practice, leaving her in good condition, that she did not attack her physically and verbally but only told her to leave the man in question alone.

# BRIEF PSYCHIATRIC RATING SCALE (BPRS) (version 4.0)

# Scoring

*Evaluate items 1-14 on the basis of what the patient says during the interview. Mark "NA" for symptoms that have not been evaluated. Note: items 7, 12 and 13 must be also coded on the basis of the behavior observed during the interview. Remember that the scale to be used is the following*

| **NA** | **1** | **2** | **3** | **4** | **5** | **6** | **7** |
| --- | --- | --- | --- | --- | --- | --- | --- |
| not assessed | not present | very mild | mild | moderate | moderately severe | severe | extremely severe |

| 1. Somatic concern | NV | 1 | 2 | 3 | 4 | 5 | 6 | 7 |  |
| --- | --- | --- | --- | --- | --- | --- | --- | --- | --- |
| 2. Anxiety | NV | 1 | 2 | 3 | 4 | 5 | 6 | 7 |  |
| 3. Depression | NV | 1 | 2 | 3 | 4 | 5 | 6 | 7 |  |
| 4. Suicidality | NV | 1 | 2 | 3 | 4 | 5 | 6 | 7 |  |
| 5. Guilt | NV | 1 | 2 | 3 | 4 | 5 | 6 | 7 |  |
| 6. Hostility | NV | 1 | 2 | 3 | 4 | 5 | 6 | 7 |  |
| 7. Elevated Mood | NV | 1 | 2 | 3 | 4 | 5 | 6 | 7 |  |
| 8. Grandiosity | NV | 1 | 2 | 3 | 4 | 5 | 6 | 7 |  |
| 9. Suspiciousness | NV | 1 | 2 | 3 | 4 | 5 | 6 | 7 |  |
| 10. Hallucinations | NV | 1 | 2 | 3 | 4 | 5 | 6 | 7 |  |
| 11. Unusual thoughts content | NV | 1 | 2 | 3 | 4 | 5 | 6 | 7 |  |
| 12. Bizarre behavior | NV | 1 | 2 | 3 | 4 | 5 | 6 | 7 |  |
| 13. Self-neglect | NV | 1 | 2 | 3 | 4 | 5 | 6 | 7 |  |
| 14. Disorientation | NV | 1 | 2 | 3 | 4 | 5 | 6 | 7 |  |

*Evaluate the following items 15 to 24 on the basis of the patient's speech and behavior during the interview.*

| 15. Conceptual disorganization | NV | 1 | 2 | 3 | 4 | 5 | 6 | 7 |  |
| --- | --- | --- | --- | --- | --- | --- | --- | --- | --- |
| 16. Blunted affect | NV | 1 | 2 | 3 | 4 | 5 | 6 | 7 |  |
| 17. Emotional withdrawal | NV | 1 | 2 | 3 | 4 | 5 | 6 | 7 |  |
| 18. Motor retardation | NV | 1 | 2 | 3 | 4 | 5 | 6 | 7 |  |
| 19. Tension | NV | 1 | 2 | 3 | 4 | 5 | 6 | 7 |  |
| 20. Uncooperativeness | NV | 1 | 2 | 3 | 4 | 5 | 6 | 7 |  |
| 21. Excitement | NV | 1 | 2 | 3 | 4 | 5 | 6 | 7 |  |
| 22. Distractibility | NV | 1 | 2 | 3 | 4 | 5 | 6 | 7 |  |
| 23. Motor hyperactivity | NV | 1 | 2 | 3 | 4 | 5 | 6 | 7 |  |
| 24. Mannerisms and posturing | NV | 1 | 2 | 3 | 4 | 5 | 6 | 7 |  |

**FORENSIC CASE N 7 – Unspecified Personality Disorder, Unspecified Bipolar Disorder**

**The crime**

The defendant is a 44-year-old woman, accused of threatening violence, personal injury, property damage and resistance to public officials. The victim reports that on the morning in question, at 11:00 am, she went with her car to shop in the supermarket. Arrived in front of the same, she was going to park in the only place left available. At this point, the defendant, who had the car pulled up nearby, got out of his car and began to inveigh against her in an intimidating way claiming the same parking. The victim, fearing for his own safety, remained aboard his car. The defendant, exiting her car, clung to that of the victim, in an attempt to extract her by force. At that moment a police patrol, alerted by some passers-by who had witnessed the scene, intervened and, in an attempt to calm the defendant, were attacked by her. Once they managed to immobilize the defendant, an ambulance was alerted for the victim who was diagnosed with 10-day prognosis in the hospital. The defendant, visited by the ambulance staff, was involuntarily hospitalized for "psychomotor agitation".

**Anamnesis**

The mother is 73 years old, she is a housewife, and she suffers from diabetes.

The father died at the age of 65 from pulmonary cancer.

She is a third child. She has a 50-year-old sister and a 47-year-old brother, both in good health, with whom she has no relationship, following disagreements over her father's inheritance.

She denies psychiatric and neurological familiarity.

Schooling: high school diploma, then she went to a professional institute. She currently works as a labor consultant.

She reports few sentimental relationships, mostly conflictual.

She reports a diagnosis of breast cancer about 4 years ago, following which psychopathological problems appear, characterized by panic attacks, attention deficits, concentration problems, depressive experiences with energy loss. Two hospitalizations in an acute psychiatric ward are reported: the first 4 years ago with the diagnosis of "Iatrogenic excitement (probably corticosteroids) in a patient in cancer therapy" and the second 6 months after the first with a diagnosis of "mood disorder - excitement". From then on, she was under treatment at the local mental health center with mood stabilizer therapy; for about 1 year she has not been taking any therapy.

**Status**

At the forensic psychiatric evaluation, the defendant is sufficiently smart in appearance and personal hygiene. She is oriented in time, space and in relation to the context.

She accesses the interview in a formally available manner, there is a tendency to describe herself with the image of a functioning person, free from mental disorders. A certain underlying suspiciousness is appreciated, there is a state of hypervigilance. No memory alterations or attention deficits emerge.

Delusions do not emerge, although there is a certain tendency to interpret various events in a tendentially persecutory and self-referenced sense. There is a certain thought acceleration, with loosening of the associative connections, limited to the phases of the most recent psychopathological decompensation. She denies current and past perceptual disturbances.

The mood is oriented in a depressive sense, with mixed aspects, in particular anger and resentment, in a context of rather marked basic humoral instability. Affectivity is characterized by a moderate level of free and somatized anxiety. Cognitive functioning is normal and of a good standard. The awareness of illness and need for psychiatric care is entirely marginal.

Regarding the crime, she reported having been robbed by the victim of the parking place she was preparing to carry out. She got out of the car for clarification, declares that she remembers that she was screaming very loudly. Then the Police arrived and, perhaps seeing her aggressive (but she was "only afraid"), put handcuffs on her and lead her to the station. In the station, she recalls that they called the ambulance, she was given an injection and then she woke up in the hospital.

# BRIEF PSYCHIATRIC RATING SCALE (BPRS) (version 4.0)

# Scoring

*Evaluate items 1-14 on the basis of what the patient says during the interview. Mark "NA" for symptoms that have not been evaluated. Note: items 7, 12 and 13 must be also coded on the basis of the behavior observed during the interview. Remember that the scale to be used is the following*

| **NA** | **1** | **2** | **3** | **4** | **5** | **6** | **7** |
| --- | --- | --- | --- | --- | --- | --- | --- |
| not assessed | not present | very mild | mild | Moderate | moderately severe | severe | extremely severe |

| 1. Somatic concern | NV | 1 | 2 | 3 | 4 | 5 | 6 | 7 |  |
| --- | --- | --- | --- | --- | --- | --- | --- | --- | --- |
| 2. Anxiety | NV | 1 | 2 | 3 | 4 | 5 | 6 | 7 |  |
| 3. Depression | NV | 1 | 2 | 3 | 4 | 5 | 6 | 7 |  |
| 4. Suicidality | NV | 1 | 2 | 3 | 4 | 5 | 6 | 7 |  |
| 5. Guilt | NV | 1 | 2 | 3 | 4 | 5 | 6 | 7 |  |
| 6. Hostility | NV | 1 | 2 | 3 | 4 | 5 | 6 | 7 |  |
| 7. Elevated Mood | NV | 1 | 2 | 3 | 4 | 5 | 6 | 7 |  |
| 8. Grandiosity | NV | 1 | 2 | 3 | 4 | 5 | 6 | 7 |  |
| 9. Suspiciousness | NV | 1 | 2 | 3 | 4 | 5 | 6 | 7 |  |
| 10. Hallucinations | NV | 1 | 2 | 3 | 4 | 5 | 6 | 7 |  |
| 11. Unusual thoughts content | NV | 1 | 2 | 3 | 4 | 5 | 6 | 7 |  |
| 12. Bizarre behavior | NV | 1 | 2 | 3 | 4 | 5 | 6 | 7 |  |
| 13. Self-neglect | NV | 1 | 2 | 3 | 4 | 5 | 6 | 7 |  |
| 14. Disorientation | NV | 1 | 2 | 3 | 4 | 5 | 6 | 7 |  |

*Evaluate the following items 15 to 24 on the basis of the patient's speech and behavior during the interview.*

| 15. Conceptual disorganization | NV | 1 | 2 | 3 | 4 | 5 | 6 | 7 |  |
| --- | --- | --- | --- | --- | --- | --- | --- | --- | --- |
| 16. Blunted affect | NV | 1 | 2 | 3 | 4 | 5 | 6 | 7 |  |
| 17. Emotional withdrawal | NV | 1 | 2 | 3 | 4 | 5 | 6 | 7 |  |
| 18. Motor retardation | NV | 1 | 2 | 3 | 4 | 5 | 6 | 7 |  |
| 19. Tension | NV | 1 | 2 | 3 | 4 | 5 | 6 | 7 |  |
| 20. Uncooperativeness | NV | 1 | 2 | 3 | 4 | 5 | 6 | 7 |  |
| 21. Excitement | NV | 1 | 2 | 3 | 4 | 5 | 6 | 7 |  |
| 22. Distractibility | NV | 1 | 2 | 3 | 4 | 5 | 6 | 7 |  |
| 23. Motor hyperactivity | NV | 1 | 2 | 3 | 4 | 5 | 6 | 7 |  |
| 24. Mannerisms and posturing | NV | 1 | 2 | 3 | 4 | 5 | 6 | 7 |  |

**FORENSIC CASE N 8 – Substance Induced Psychotic Disorder in Paranoid Personality Disorder**

**The crime**

The defendant is a 32-year-old man who was sent to prison for killing his uncle with 8 downward blows in the chest. The defendant's father reported that a few days before the crime, the defendant allegedly expressed concerns about possible death threats against his family.

**Anamnesis**

The father is 60 years-old, he is in treatment for hypertension.

The mother is 55 years-old and she is in apparent good health.

He is the second of three children. A 34-year-old brother suffers from Schizophrenia, while 26-year -old sister is in apparent good health.

Schooling: high school diploma. He was rejected in first year of high school. He is currently enrolled in the Faculty of Chemistry. Sufficient academic performance is reported.

He reports having had two emotional relationships in the past, of which however he does not provide further explanations.

Since adolescence, use of cannabinoids has been reported, initially occasional, in recent years daily.

**Status**

At the forensic psychiatric evaluation, the defendant appears poorly cared for in appearance and personal hygiene. He is oriented in time, space and in relation to the context. The gaze supports that of the examiner, although the mimicry is not always congruous with the issues addressed. The attitude is hypervigil.

There is a delusional ideation with a persecutory background towards the victim, who is believed to have hurt the defendant’s family. He is convinced that his brother has previously attacked the victim and that in order to take revenge, the victim has made his brother mad, to the point of making him suffer from schizophrenia. He reports that he spoke to his father about his problems with the victim several times before the crime, but the father wanted him to take the medicine and visit the psychiatrist who followed his brother, but that he was against it.

He also went to talk to the victim days before the crime, to convince him to leave his family alone, but it hadn’t helped. He therefore reports that on the evening in question he went to the victim's home on the pretext of speaking and that he killed him. After the incident, he returned home, confessed the crime to his parents and then called the police to be arrested. There are no reports of past or current perceptual disturbances.

The affectivity is flattened, the mood is oriented in a depressive sense.

Illness awareness is poor.

# BRIEF PSYCHIATRIC RATING SCALE (BPRS) (version 4.0)

# Scoring

*Evaluate items 1-14 on the basis of what the patient says during the interview. Mark "NA" for symptoms that have not been evaluated. Note: items 7, 12 and 13 must be also coded on the basis of the behavior observed during the interview. Remember that the scale to be used is the following*

| **NA** | **1** | **2** | **3** | **4** | **5** | **6** | **7** |
| --- | --- | --- | --- | --- | --- | --- | --- |
| not assessed | not present | very mild | mild | moderate | moderately severe | severe | extremely severe |

| 1. Somatic concern | NV | 1 | 2 | 3 | 4 | 5 | 6 | 7 |  |
| --- | --- | --- | --- | --- | --- | --- | --- | --- | --- |
| 2. Anxiety | NV | 1 | 2 | 3 | 4 | 5 | 6 | 7 |  |
| 3. Depression | NV | 1 | 2 | 3 | 4 | 5 | 6 | 7 |  |
| 4. Suicidality | NV | 1 | 2 | 3 | 4 | 5 | 6 | 7 |  |
| 5. Guilt | NV | 1 | 2 | 3 | 4 | 5 | 6 | 7 |  |
| 6. Hostility | NV | 1 | 2 | 3 | 4 | 5 | 6 | 7 |  |
| 7. Elevated Mood | NV | 1 | 2 | 3 | 4 | 5 | 6 | 7 |  |
| 8. Grandiosity | NV | 1 | 2 | 3 | 4 | 5 | 6 | 7 |  |
| 9. Suspiciousness | NV | 1 | 2 | 3 | 4 | 5 | 6 | 7 |  |
| 10. Hallucinations | NV | 1 | 2 | 3 | 4 | 5 | 6 | 7 |  |
| 11. Unusual thoughts content | NV | 1 | 2 | 3 | 4 | 5 | 6 | 7 |  |
| 12. Bizarre behavior | NV | 1 | 2 | 3 | 4 | 5 | 6 | 7 |  |
| 13. Self-neglect | NV | 1 | 2 | 3 | 4 | 5 | 6 | 7 |  |
| 14. Disorientation | NV | 1 | 2 | 3 | 4 | 5 | 6 | 7 |  |

*Evaluate the following items 15 to 24 on the basis of the patient's speech and behavior during the interview.*

| 15. Conceptual disorganization | NV | 1 | 2 | 3 | 4 | 5 | 6 | 7 |  |
| --- | --- | --- | --- | --- | --- | --- | --- | --- | --- |
| 16. Blunted affect | NV | 1 | 2 | 3 | 4 | 5 | 6 | 7 |  |
| 17. Emotional withdrawal | NV | 1 | 2 | 3 | 4 | 5 | 6 | 7 |  |
| 18. Motor retardation | NV | 1 | 2 | 3 | 4 | 5 | 6 | 7 |  |
| 19. Tension | NV | 1 | 2 | 3 | 4 | 5 | 6 | 7 |  |
| 20. Uncooperativeness | NV | 1 | 2 | 3 | 4 | 5 | 6 | 7 |  |
| 21. Excitement | NV | 1 | 2 | 3 | 4 | 5 | 6 | 7 |  |
| 22. Distractibility | NV | 1 | 2 | 3 | 4 | 5 | 6 | 7 |  |
| 23. Motor hyperactivity | NV | 1 | 2 | 3 | 4 | 5 | 6 | 7 |  |
| 24. Mannerisms and posturing | NV | 1 | 2 | 3 | 4 | 5 | 6 | 7 |  |

**FORENSIC CASE N 9 – Epilepsy –Jacksonian motoric crises, right facial-brachial-crural type with secondary generalization**

**The crime**

The defendant is a 41-year-old man who was sent to prison for the killing by means of 18 stab wounds of Mr. A. M., during the execution, together with his brother-in-law, of a robbery in a jewelry store.

**Anamnesis**

The father died at the age of 60 because of lung cancer.

The mother is 65 years-old m and she is in apparent good health

He is the third of three children. He has two brothers aged 45 and 43, respectively, both in apparent good health.

Schooling: high school diploma. He reports good functioning up to the age of 21, the year in which a road accident is reported, during which he lost consciousness and reported a head injury. Since then, dizziness, clonic shocks to the right arm, sometimes with loss of consciousness and memory problems appeared. The diagnosis for this symptomatology was: "Epilepsy –Jacksonian motoric crises, right facial-brachial-crural type with secondary generalization ". He had a handicap recognition for this neurological condition, and he was without work for a long time, then he worked as a bricklayer. He reports several romantic relationships, one of which lasted for about 8 years with a peer with whom he lived with, interrupted 3 years ago at her behest.

**Status**

At the forensic psychiatric evaluation appears smart in appearance and personal hygiene. The state of consciousness is alert, oriented over time, in space and towards the examiner There are no anomalies in concentration and memory, although the subject reports that the latter is not always effective, having had episodes in which he found himself in places unknown to him without knowing how he got there. Mimicry is mobile and appropriate to the issues addressed; the speech is fluid.

Formal logical disturbances of thought and perception are absent.

There is a mild depressive condition. On the whole, his personality seems to be characterized by elements of superficiality, passivity and dependence. Mild free anxiety emerges.

Regarding the crime, the defendant stubbornly denies his involvement in the murder, stating that he went to the place in the car with his brother-in-law and stayed in the car to wait. He reports that he had knowledge of what he was going to do in terms of robbery, but that he was very scared and was practically forced by his brother-in-law, a person with alcohol abuse and violent problems, because they had economic difficulties. Subsequently, his brother-in-law jumped into the car and told him that they had to escape because there had been a "problem".

Instrumental exams

EEG: normal

MRI with contrast medium: absence of pathological elements even after administration of contrast medium

# BRIEF PSYCHIATRIC RATING SCALE (BPRS) (version 4.0)

# Scoring

*Evaluate items 1-14 on the basis of what the patient says during the interview. Mark "NA" for symptoms that have not been evaluated. Note: items 7, 12 and 13 must be also coded on the basis of the behavior observed during the interview. Remember that the scale to be used is the following*

| **NA** | **1** | **2** | **3** | **4** | **5** | **6** | **7** |
| --- | --- | --- | --- | --- | --- | --- | --- |
| not assessed | not present | very mild | mild | moderate | moderately severe | severe | extremely severe |

| 1. Somatic concern | NV | 1 | 2 | 3 | 4 | 5 | 6 | 7 |  |
| --- | --- | --- | --- | --- | --- | --- | --- | --- | --- |
| 2. Anxiety | NV | 1 | 2 | 3 | 4 | 5 | 6 | 7 |  |
| 3. Depression | NV | 1 | 2 | 3 | 4 | 5 | 6 | 7 |  |
| 4. Suicidality | NV | 1 | 2 | 3 | 4 | 5 | 6 | 7 |  |
| 5. Guilt | NV | 1 | 2 | 3 | 4 | 5 | 6 | 7 |  |
| 6. Hostility | NV | 1 | 2 | 3 | 4 | 5 | 6 | 7 |  |
| 7. Elevated Mood | NV | 1 | 2 | 3 | 4 | 5 | 6 | 7 |  |
| 8. Grandiosity | NV | 1 | 2 | 3 | 4 | 5 | 6 | 7 |  |
| 9. Suspiciousness | NV | 1 | 2 | 3 | 4 | 5 | 6 | 7 |  |
| 10. Hallucinations | NV | 1 | 2 | 3 | 4 | 5 | 6 | 7 |  |
| 11. Unusual thoughts content | NV | 1 | 2 | 3 | 4 | 5 | 6 | 7 |  |
| 12. Bizarre behavior | NV | 1 | 2 | 3 | 4 | 5 | 6 | 7 |  |
| 13. Self-neglect | NV | 1 | 2 | 3 | 4 | 5 | 6 | 7 |  |
| 14. Disorientation | NV | 1 | 2 | 3 | 4 | 5 | 6 | 7 |  |

*Evaluate the following items 15 to 24 on the basis of the patient's speech and behavior during the interview.*

| 15. Conceptual disorganization | NV | 1 | 2 | 3 | 4 | 5 | 6 | 7 |  |
| --- | --- | --- | --- | --- | --- | --- | --- | --- | --- |
| 16. Blunted affect | NV | 1 | 2 | 3 | 4 | 5 | 6 | 7 |  |
| 17. Emotional withdrawal | NV | 1 | 2 | 3 | 4 | 5 | 6 | 7 |  |
| 18. Motor retardation | NV | 1 | 2 | 3 | 4 | 5 | 6 | 7 |  |
| 19. Tension | NV | 1 | 2 | 3 | 4 | 5 | 6 | 7 |  |
| 20. Uncooperativeness | NV | 1 | 2 | 3 | 4 | 5 | 6 | 7 |  |
| 21. Excitement | NV | 1 | 2 | 3 | 4 | 5 | 6 | 7 |  |
| 22. Distractibility | NV | 1 | 2 | 3 | 4 | 5 | 6 | 7 |  |
| 23. Motor hyperactivity | NV | 1 | 2 | 3 | 4 | 5 | 6 | 7 |  |
| 24. Mannerisms and posturing | NV | 1 | 2 | 3 | 4 | 5 | 6 | 7 |  |

**FORENSIC CASE N 10 – Borderline intellectual functioning and Other Specified Personality Disorder, mixed personality features (Borderline/Antisocial) with impulsive sadistic traits related to sexual themes, probably paraphilic**

**The crime**

The accused is a 49-year-old man confined in prison for attacking and attempting to kill a prostitute by means of a knife, with 3 downward blows to the abdomen.

**Anamnesis**

The father is 75 years-old, he is under treatment for hypertension and has type II diabetes.

The mother is 71-years-old, she is affected by type II diabetes.

He is the eldest of two children: his sister, 8 years younger, is in apparent good health. Schooling: lower secondary school. He then carried out various work activities (bricklayer, haulier), never managing to maintain a stable job. Since 2010 he has a handicap recognition for "antisocial personality disorder with hetero aggressive behavior". He never had meaningful romantic relationships, nor did he actually have friendly relationships. Most of the relationships with the other sex are described with prostitutes, not exclusively for sexual purposes, and the sexual ones also with multiple partners simultaneously.

He reports previous alcohol abuse, although he claims to be abstinent in the last period. He has been subjected to several convictions for crimes against the person, property and violation of the arms law. He reports in 2010 that he was interned in a forensic facility for a crime similar to the current one, for which he had been judged not accountable for "schizophrenic psychotic syndrome developed within a limit intellectual level and a disturbed basic personality, and socially dangerous from a psychiatric point of view ". He declares that he has never been treated from a psychiatric point of view, with the exception of the forensic facility.

**Status**

At the forensic psychiatric evaluation, he appears quite smart in appearance. The state of consciousness is quantitatively alert; on a qualitative level, he appeared oriented over time, in space and towards the examiner, whose role he understood well. A detached, elusive attitude emerges on many subjects, at times he is frankly reticent and oppositional, probably because of, at least in part, a basic suspiciousness of the defendant.

Specifically, the defendant alleges memory problems especially when asked to express himself on topics pertaining to the accusations of which he is subject, with respect to which he claimed to consider himself innocent because he was provoked.

The mimicry is rigid and expressionless; the speech is fluid, non-spontaneous, it constantly needs to be elicited with questions.

The thought seems rather poor and concrete, affective participation is scarce.

There are no current esplicit formal logical alterations of thought or of perception. However, there is an underlying suspicion, widespread with a certain tendency towards interpretation in the sense of self-reference. Judgment, criticism and forecasting skills are sufficiently conserved. A low propensity to adapt to the usual social rules emerges, together with a tendency to be pleased with the suffering of others. Invited to describe his experiences in the various circumstances in which in the past he found himself exercising violent acts on women, he said he felt pleasure and anger at the same time. Absent from free and somatized anxiety.

Regarding to the crime in question, he reconstructed the episode with clarity, claiming that he had been lured in a bar by the victim, who had invited him to have sex with her. Initially contrary, at the insistence of the girl he followed her to a secluded place together. In that place he later changed his mind and pushed the girl in an attempt to push her away; the latter in turn bit him on the hand, triggering a strong reaction of anger in him ("I saw red") which then led to the crime in question.

# BRIEF PSYCHIATRIC RATING SCALE (BPRS) (version 4.0)

# Scoring

*Evaluate items 1-14 on the basis of what the patient says during the interview. Mark "NA" for symptoms that have not been evaluated. Note: items 7, 12 and 13 must be also coded on the basis of the behavior observed during the interview. Remember that the scale to be used is the following*

| **NA** | **1** | **2** | **3** | **4** | **5** | **6** | **7** |
| --- | --- | --- | --- | --- | --- | --- | --- |
| not assessed | not present | very mild | mild | moderate | moderately severe | severe | extremely severe |

| 1. Somatic concern | NV | 1 | 2 | 3 | 4 | 5 | 6 | 7 |  |
| --- | --- | --- | --- | --- | --- | --- | --- | --- | --- |
| 2. Anxiety | NV | 1 | 2 | 3 | 4 | 5 | 6 | 7 |  |
| 3. Depression | NV | 1 | 2 | 3 | 4 | 5 | 6 | 7 |  |
| 4. Suicidality | NV | 1 | 2 | 3 | 4 | 5 | 6 | 7 |  |
| 5. Guilt | NV | 1 | 2 | 3 | 4 | 5 | 6 | 7 |  |
| 6. Hostility | NV | 1 | 2 | 3 | 4 | 5 | 6 | 7 |  |
| 7. Elevated Mood | NV | 1 | 2 | 3 | 4 | 5 | 6 | 7 |  |
| 8. Grandiosity | NV | 1 | 2 | 3 | 4 | 5 | 6 | 7 |  |
| 9. Suspiciousness | NV | 1 | 2 | 3 | 4 | 5 | 6 | 7 |  |
| 10. Hallucinations | NV | 1 | 2 | 3 | 4 | 5 | 6 | 7 |  |
| 11. Unusual thoughts content | NV | 1 | 2 | 3 | 4 | 5 | 6 | 7 |  |
| 12. Bizarre behavior | NV | 1 | 2 | 3 | 4 | 5 | 6 | 7 |  |
| 13. Self-neglect | NV | 1 | 2 | 3 | 4 | 5 | 6 | 7 |  |
| 14. Disorientation | NV | 1 | 2 | 3 | 4 | 5 | 6 | 7 |  |

*Evaluate the following items 15 to 24 on the basis of the patient's speech and behavior during the interview.*

| 15. Conceptual disorganization | NV | 1 | 2 | 3 | 4 | 5 | 6 | 7 |  |
| --- | --- | --- | --- | --- | --- | --- | --- | --- | --- |
| 16. Blunted affect | NV | 1 | 2 | 3 | 4 | 5 | 6 | 7 |  |
| 17. Emotional withdrawal | NV | 1 | 2 | 3 | 4 | 5 | 6 | 7 |  |
| 18. Motor retardation | NV | 1 | 2 | 3 | 4 | 5 | 6 | 7 |  |
| 19. Tension | NV | 1 | 2 | 3 | 4 | 5 | 6 | 7 |  |
| 20. Uncooperativeness | NV | 1 | 2 | 3 | 4 | 5 | 6 | 7 |  |
| 21. Excitement | NV | 1 | 2 | 3 | 4 | 5 | 6 | 7 |  |
| 22. Distractibility | NV | 1 | 2 | 3 | 4 | 5 | 6 | 7 |  |
| 23. Motor hyperactivity | NV | 1 | 2 | 3 | 4 | 5 | 6 | 7 |  |
| 24. Mannerisms and posturing | NV | 1 | 2 | 3 | 4 | 5 | 6 | 7 |  |
